# Saturated linkage map construction in *Rubus idaeus* using genotyping by sequencing and genome-independent imputation

**DOI:** 10.1186/1471-2164-14-2

**Published:** 2013-01-16

**Authors:** Judson A Ward, Jasbir Bhangoo, Felicidad Fernández-Fernández, Patrick Moore, JD Swanson, Roberto Viola, Riccardo Velasco, Nahla Bassil, Courtney A Weber, Daniel J Sargent

**Affiliations:** 1 Department of Horticulture, Cornell University, New York State Agricultural Experiment Station, Geneva, New York, 14456, USA; 2 Sector 18, Chandigarh, UT, 160018, India; 3 East Malling Research (EMR), New Road, East Malling, Kent, ME, 19 6BJ, UK; 4 Department of Horticulture and Landscape Architecture, Washington State University Puyallup Research and Extension Center, Puyallup, WA, 98372, USA; 5 Department of Biology and Biomedical Sciences, Salve Regina University, 100 Ochre Point Ave, Newport, RI, 02840, USA; 6 IASMA Research and Innovation Centre, Foundation Edmund Mach, Via E Mach 1, San Michele all’Adige, (TN), 38010, Italy; 7 USDA-ARS National Clonal Germplasm Repository, 33447 Peoria Rd, Corvallis, OR, 97333, USA

**Keywords:** Genotyping by sequencing, GBS, RADseq, Imputation, Raspberry, Rubus idaeus, Psuedotestcross, Linkage map, Segregation distortion

## Abstract

**Background:**

Rapid development of highly saturated genetic maps aids molecular breeding, which can accelerate gain per breeding cycle in woody perennial plants such as *Rubus idaeus* (red raspberry). Recently, robust genotyping methods based on high-throughput sequencing were developed, which provide high marker density, but result in some genotype errors and a large number of missing genotype values. Imputation can reduce the number of missing values and can correct genotyping errors, but current methods of imputation require a reference genome and thus are not an option for most species.

**Results:**

Genotyping by Sequencing (GBS) was used to produce highly saturated maps for a *R. idaeus* pseudo-testcross progeny. While low coverage and high variance in sequencing resulted in a large number of missing values for some individuals, a novel method of imputation based on maximum likelihood marker ordering from initial marker segregation overcame the challenge of missing values, and made map construction computationally tractable. The two resulting parental maps contained 4521 and 2391 molecular markers spanning 462.7 and 376.6 cM respectively over seven linkage groups. Detection of precise genomic regions with segregation distortion was possible because of map saturation. Microsatellites (SSRs) linked these results to published maps for cross-validation and map comparison.

**Conclusions:**

GBS together with genome-independent imputation provides a rapid method for genetic map construction in any pseudo-testcross progeny. Our method of imputation estimates the correct genotype call of missing values and corrects genotyping errors that lead to inflated map size and reduced precision in marker placement. Comparison of SSRs to published *R. idaeus* maps showed that the linkage maps constructed with GBS and our method of imputation were robust, and marker positioning reliable. The high marker density allowed identification of genomic regions with segregation distortion in *R. idaeus*, which may help to identify deleterious alleles that are the basis of inbreeding depression in the species.

## Background

Genetic linkage maps permit the elucidation of genome structure and organization and enable the identification of molecular markers linked to traits in an experimental segregating progeny, leading ultimately to the elucidation of the genetic basis of the trait of interest. As a result, maps have been developed for many diverse plant species [1-9]. Traditionally, transferable linkage map development has been achieved through the scoring of restriction fragment length polymorphisms (RFLPs) [5], microsatellites (SSRs) and gene specific markers [3] in a segregating progeny. Using such markers, saturated reference linkage maps for many plant species have been developed. Reference maps inform the selection of markers for mapping in other progenies [10-12] and have been used to anchor, order and orientate physical map BAC contigs, and genome sequencing scaffolds for the assignment of pseudo-chromosomes for whole genome sequence initiatives [13-17].

Single nucleotide polymorphisms (SNPs) are the most abundant mutations between related DNA molecules. The advent of affordable second generation sequencing technologies in recent years has led to the release of whole genome reference sequences for many plant species [6,14,18-20], and the identification of abundant SNPs throughout the genomes of these organisms [21-23]. Thus, SNPs are becoming increasingly important as markers for both fundamental and applied genetics research in plants. Relatively low throughput methods have been developed for the analysis and mapping of SNPs. These include high resolution melting (HRM) [24], and the cleaved amplified polymorphic DNA (CAPs) assay [25]. Additionally, medium and high throughput genotyping assays have been developed that permit hundreds of thousands of SNPs to be interrogated simultaneously on a single multiplexed array. Platforms for genotyping in this way include SNPlex, Golden Gate, Infinium and Axiom, which have been employed successfully for genotyping in many plant species including apple, peach, grape and purple false brome [22,23,26-29]. Genotyping arrays have many advantages over other techniques for genetic analysis, however an essential prerequisite for array development is a predetermined set of SNPs, preferably located at known positions on a reference genome sequence. Additionally, the transferability of heterozygous SNPs between species has been shown to be low [30] and as such, in many genera, arrays must be developed specifically for the species under investigation. Thus for minor crops and for genotyping interspecific progenies or species complexes, the development of arrays is currently not a viable experimental solution.

Despite the second generation sequencing ‘revolution’ in the biological sciences, many crops of significant economic importance remain without a reference genome sequence, or an abundance of SNP data in public repositories. High throughput SNP genotyping for these organisms using array-based technologies is not economically viable, yet rapid, high-throughput SNP genotyping would be immensely advantageous for the progression of classical mapping and QTL analyses, for genome-wide association studies and pedigree-based analyses, genomic selection and for the development and implementation of marker-assisted breeding and selection.

Second generation sequencing has offered the possibility to genotype sequence variation in the genome of an organism for use in mapping experiments through whole genome re-sequencing. Whole genome re-sequencing has been employed for mapping in eukaryotic species with a relatively small genome size and on a selective mapping populations such as for the fungus *Venturia inequalis*[21]*.* However, for the majority of organisms, even those with relatively small genomes such as the diploid strawberry *Fragaria vesca*[14,21] a complexity reduction step must be performed prior to sequencing to enable sufficient depth of coverage of the same regions in all genomes of a segregating progeny to permit segregation to be scored. Genotyping through the sequencing of reduced representation genomic libraries developed through restriction digestion of genomic DNA (restriction-site associated DNA; RAD) was initially proposed by Miller (2007) [31] and adapted to incorporate barcoding for multiplexing with Illumina sequencing technology by Baird et al. (2008) [32]. The RAD procedure has been used successfully to identify SNPs in a number of plant species including eggplant, barley, and globe artichoke [33-35] and its utility to linkage map development and QTL analysis in a large mapping population was demonstrated recently by Pfender et al. (2011) [36]. Subsequently, Elshire et al. (2011**)**[37] proposed a method for the construction of highly multiplexed reduced complexity genotyping by sequencing (GBS) libraries. The procedure is based on a similar restriction digestion technique to RAD, however it is substantially less complicated, resulting in time and cost savings in library preparation, but the resultant data contains a larger number of missing genotype calls.

*Rubus* is a genus in the Rosaceae family containing more than 600 species, some of which, such as *R. idaeus* subsp. *idaeus* L. (red raspberry) and *Rubus* L. subgenus *Rubus* Watson (blackberry) are of economic importance as cultivated fruit crops. Breeding methods for these species have remained largely unchanged since the first empirical breeding programs were initiated. However, changes in cultural practices, the withdrawl of soil fumigants, and demands for increased fruit quality, shelf-life and for the extension of the traditional cultivation season, have necessitated novel breeding techniques to satisfy the demand for new cultivars. The development and application of molecular tools for *Rubus* would increase the speed and precision of the breeding process, particularly for traits that are difficult to characterize phenotypically, such as pyramided resistances to pests or pathogens. Looking further forward, *Rubus* breeding would greatly benefit from genomic selection approaches that have recently become popular in crops such as maize, barley, and wheat [38] because even modest gains from genomic selection could save years of in-field evaluation. An essential precursor to the development of such tools is the characterization of an abundance of informative molecular markers with which to perform marker-trait association analyses. In *Rubus*, the majority of molecular markers that have been developed and mapped in the genus to date are SSRs [4,39-43]. More recently, low throughput methods were employed for mapping SNP markers in an interspecific *Rubus* mapping progeny [44,45], but high throughput methods for the identification and mapping of molecular markers have yet to be reported for the genus.

In this investigation, we have exploited the recent advances in low-cost sequencing and multiplexed library preparation [37] to generate segregation data for SNP markers distributed throughout the *R. idaeus* genome. We used these markers for linkage map construction in a red raspberry progeny from the cross ‘Heritage’×‘Tulameen’ (H×T). The segregation data was generated using multiplexed sequencing on the Illumina HiSeq sequencing-by-synthesis platform. Shallow genome sampling resulted in a data set containing a large proportion of missing values, and thus we developed a pipeline which includes a novel imputation algorithm (Maskov) to deal with the missing and putatively erroneous data through comparison of genotypes in internal genotype bins following initial co-segregation analysis. The challenges and solutions to generating and handling segregation data from thousands of loci for linkage mapping are discussed.

## Results

### Genotyping by sequencing

Sequencing resulted in 135,776,036 reads including deeper coverage of parents. There were 19,623,392 reads for ‘Heritage’ and 20,293,782 for ‘Tulameen’. Within the population the mean number of reads was 1,350,125 and the median was 977,402 per individual. Forty-two individuals from the progeny were sequenced in library one and 29 individuals were sequenced in library two although each individual within a library was part of a 96-plex reaction in a single sequencing lane at two different sequencing centers. Sequence quality differed between the two sequencing lanes with a mean phred score at base 64 of 26.7 in library one and a mean phred score at base 64 of 33.6 in library two. Overall, library one had lower per base quality scores and a greater per base interquartile range compared to library two (Additional file 1: Figure S1). However, on a per read basis both libraries had quality scores greater than 37 for most reads (Additional file 2: Figure S2). In library one approximately 19.15 percent of reads contained N’s (uncalled bases) compared to only 7.53 percent of reads in library two. Further differences in the error rate between the lanes is illustrated by the percentage of unique reads in each library because as coverage increases additional unique reads are likely to result from sequencing errors rather than new sequences (Additional file 3: Figure S3). Overrepresented sequences in the libraries had high sequence similarity to the *Fragaria vesca* chloroplast genome and accounted for approximately 5.5 percent of library one and approximately 6.3 percent of library two (Additional file 4: Table S1) as determined by alignment with bowtie (Langmead et al., 2009). The percent missing data was also a clear function of sequencing depth (Additional file 5: Figure S4).

### Number of segregating SNPs identified

A total of 9143 segregating SNPs were identified in the progeny following analysis of raw data using Stacks [46]. Of these, 4744 were present in the parental configuration AB×AA (i.e. heterozygous only in ‘Heritage’), 2672 in the configuration AA×AB (i.e. heterozygous only in ‘Tulameen’), and the remaining 1727 in the configuration AB×AB (i.e. heterozygous in both parents). To simplify the process of imputation, and subsequent analysis using maximum likelihood implemented in JOINMAP 4.0 (Kyasma, NL), only SNPs segregating in a uni-parental configuration, i.e. AB×AA or AA×AB were used for further analysis.

### Segregating SSRs identified in the H × T progeny

**Figure 1:**
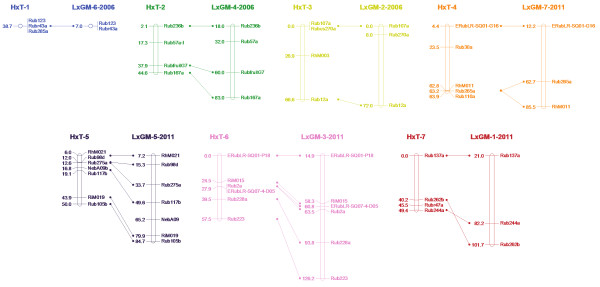
**A comparison of common markers mapped between the ‘Heritage’ linkage map in this investigation and the ‘Latham’ × ‘Glen Moy’ (L×GM) linkage maps of Graham et al. (2006, 2011)[**[42]**,**[45]**].** The version of the L×GM map that was used for comparison of each linkage group was based on the number of common markers mapped to the ‘Heritage’ map and is designated after the LG name by the year of publication. Linkage group nomenclature on the ‘Heritage’ map follows the revisions proposed by Bushakra et al. (2012) [44]. Genetic distances are given in centi-Morgans.

In total, 33 *Rubus* SSR loci, distributed throughout the seven linkage groups of previously published *Rubus* linkage maps were tested and identified as heterozygous in the ‘Heritage’ parental genotype. Of these loci, 12 were also heterozygous in the ‘Tulameen’ parental genotype. The 33 markers were scored and mapped in the H×T progeny. No heterozygous markers were found to define linkage group LG7 of the ‘Tulameen’ map, however the other six linkage groups were all assigned numbers based on the SSRs which were located to each group, and thus LG7 was defined by default. The positions of the SSRs on the ‘Heritage’ linkage map were compared to their positions on the previously published ‘Latham’ × ‘Glen Moy’ (L×GM) linkage maps of Graham et al. (2006, 2011). The version of the L×GM linkage groups containing the greatest number of common markers were compared to the ‘Heritage’ linkage map. The comparison of common markers (Figure 1) demonstrated a high degree of colinearity in marker order between the ‘Heritage’ and L×GM linkage maps. In all but one linkage group (LG7, where the position of two markers was inverted between the maps), marker order was maintained between the two populations, however, in the L×GM linkage map, genetic distances between markers were uniformly greater than on the ‘Heritage’ linkage map.

### Total number of SNPs mapped and percentage of missing values

Following initial co-segregation analysis a total of 4521 SNPs displaying the parental configuration AB × AA (i.e. heterozygous in the ‘Heritage’ parental genotype), along with the 33 SSR markers scored in the progeny, coalesced into seven linkage groups associated with the haploid chromosome number for the species at a minimum LOD score of 7.0. A further 2391 SNPs, along with 12 SSR loci displaying the parental configuration AA × AB (i.e. heterozygous in the ‘Tulameen’ parental genotype), coalesced into seven linkage groups at a minimum LOD score of 7.0. The total number of data-points analysed in the initial phase of mapping was 323,334 in 71 seedlings in the ‘Heritage’ data set and 170,613 in 71 seedlings in the ‘Tulameen’ data set, containing a total of 116,728 (36%) and 61,481 (36%) missing values respectively. The average number of perceived recombination events per individual was 22.45 in ‘Heritage’ and 11.22 in ‘Tulameen’, indicating a large number of double recombination events due to erroneous marker genotypes.

### Imputation

**Figure 2:**
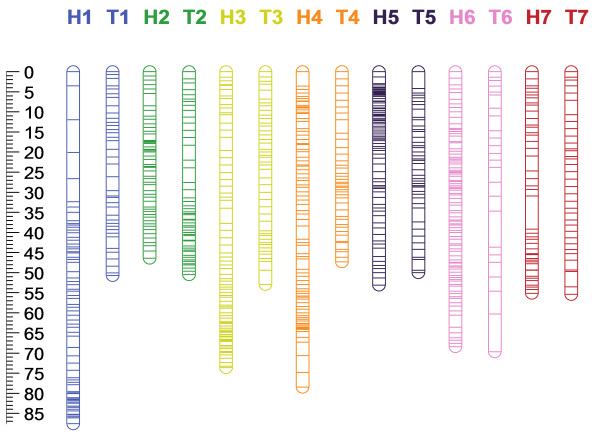
**Single nucleotide polymorphism based genetic linkage maps for ‘Heritage’ and ‘Tulameen’ composed of 4554 and 2403 molecular markers respectively, including 4521 and 2391 SNPs and 33 and 12 SSRs spanning 462.7 and 376.6 cM respectively over seven LGs.** ‘Heritage’ LGs are denoted H1-H7 and ‘Tulameen’ LGs are denoted T1-T7. The scale in centi-Morgans is given at the edge of the figure. Lines represent the positions of marker bins on each linkage group.

Following imputation of missing values and removal of suspected erroneous genotypes using Maskov, an average of 6.5 and 3.7 recombination events per individual were observed in the ‘Heritage’ and ‘Tulameen’ maps; a reduction in recombination of 71.1% and 67% respectively, whilst the total map length was reduced by 70.8% and 68.3% respectively. The 4554 and 2403 markers, including SSRs that mapped to the seven ‘Heritage’ and ‘Tulameen’ linkage groups were contained in 502 genotype bins on the ‘Heritage’ linkage map and 274 on the ‘Tulameen’ linkage map. Following co-segregation analysis using JOINMAP 4.0 (Kyasma, NL) implementing the Kosambi mapping function, the linkage maps presented in Figure 2 were resolved. The ‘Heritage’ linkage map spanned a total of 462.7 cM, with a maximum and minimum linkage group length of 87.6 cM (LG1) and 46.4 cM (LG2) respectively, an average length of 66.1 cM and an average marker density of 0.1 cM per marker, or 0.92 cM per marker based on the genotyping bins (i.e. non-identical genotypes). The ‘Tulameen’ map spanned a total of 376.6 cM with a maximum and minimum linkage group length of 69.7 cM (LG6) and 47.3 cM (LG1), an average length of 53.8 cM and an average marker density of 0.16 cM per marker, or 1.4 cM per marker based on the number of genotyping bins, rather than the total number of markers mapped. Tables 1 and 2 detail the marker composition and lengths of the seven ‘Heritage’ and ‘Tulameen’ LGs. Recombination ‘cold-spots’ were observed on six ‘Heritage’ (LG1, 2, 3, 4, 5 and 6) and one ‘Tulameen’ (LG2) linkage groups at which marker density was significantly higher than across the rest of the linkage group. The markers mapped to the two parental maps, along with the linkage group and map position to which they were located, the SNP type and the nucleotide sequence flanking each side of each SNP are detailed in Additional file 6: Table S2. Comparison of data imputed manually and that imputed using Maskov revealed that the two methods produced very similar datasets (data not shown). Imputation using Maskov tended to be more conservative due to the stringent parameters used in the pipeline. The maps produced using both methods were colinear, however, those produced using Maskov had fewer genotyping bins, containing relatively more markers, and thus had lower overall resolution than those produced manually.

**Table 1:** The total number of SNP and SSR markers mapped to the ‘Heritage’ linkage map, the number of markers per chromosome and the total length of each LG in centi-Morgans (cM)

**LG**	**LG length (cM)**	**No. SNPs**	**No. SSRs**	**No. Bins**
1	87.6	488	3	67
2	46.4	427	4	55
3	73.6	645	4	75
4	78.5	616	5	81
5	53.1	656	7	76
6	68.4	1218	6	89
7	55.1	471	4	44
Total	462.7	4521	33	487

**Table 2:** The total number of SNP and SSR markers mapped to the ‘Tulameen’ linkage map, the number of markers per chromosome and the total length of each LG in centi-Morgans (cM)

**LG**	**LG length (cM)**	**No. SNPs**	**No. SSRs**	**No. Bins**
1	50.7	348	2	38
2	50.5	493	3	49
3	52.9	311	1	44
4	47.3	450	2	40
5	50	350	3	44
6	69.7	133	1	24
7	55.5	306	0	35
Total	376.6	2391	12	274

### Segregation distortion

**Figure 3:**
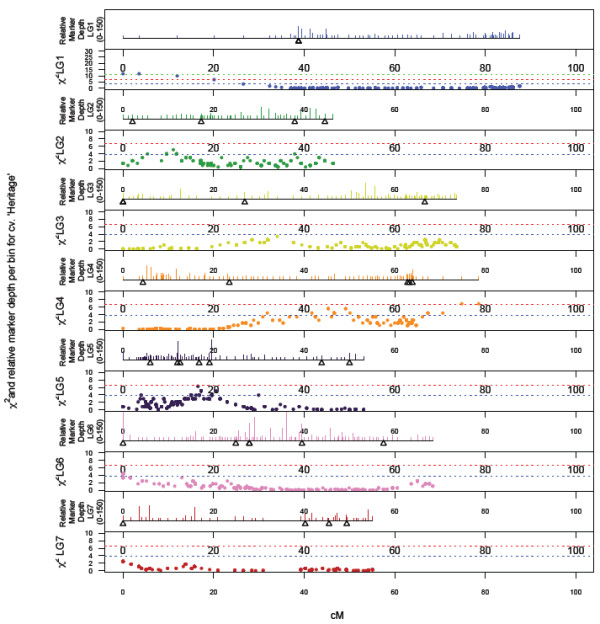
**Genome-wide patterns of marker density/depth and segregation distortion, plotted as a function of Chi-squared value for monogenic marker segregation ratios against marker position on the each of the seven LGs of the ‘Heritage’ map.** Dashed lines indicate Chi-squared significance values of P ≤ 0.05, P ≤ 0.01 and P ≤ 0.01. The scale in the upper panel of each LG is from 1 to 150 markers.

**Figure 4:**
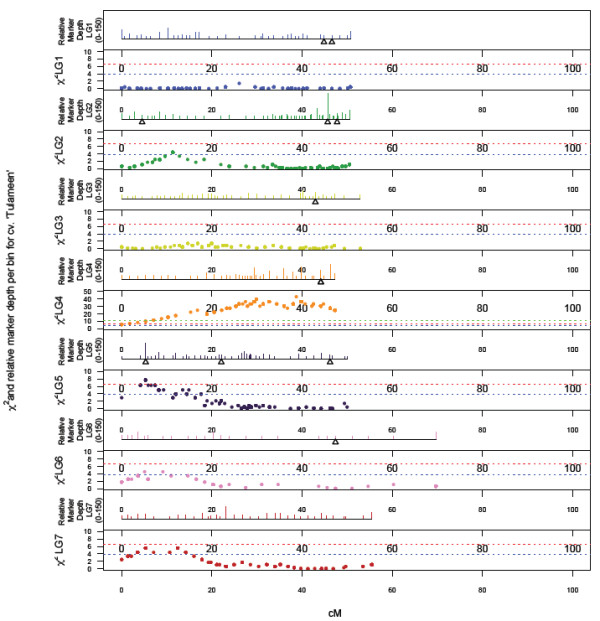
**Genome-wide patterns of marker density/depth and segregation distortion, plotted as a function of Chi-squared value for monogenic marker segregation ratios against marker position on the each of the seven LGs of the ‘Tulameen’ map.** Dashed lines indicate Chi-squared significance values of P ≤ 0.05, P ≤ 0.01 and P ≤ 0.01. The scale in the upper panel of each LG is from 1 to 150 markers.

Genome wide patterns of segregation distortion for ‘Heritage’ and ‘Tulameen’ are presented in Figures 3 and 4. A total of 345 (7.6%) SNPs mapped on the ‘Heritage’ and 653 (27.3%) SNPs mapped on the ‘Tulameen’ linkage maps displayed significant (P=>0.05) segregation distortion. Segregation distortion was non-random across the two linkage maps, with similar localized regions of distortion observed on LG2, 4, 5 and 6 of both maps. The most significant distortion was observed on LG4 of the ‘Tulameen’ linkage map, where all markers displayed highly significant segregation distortion (P=>0.001) and the LG length was significantly shorter than the other six LGs.

## Discussion

Using a recently reported method of multiplexed, reduced representation library construction [37] and massively parallel sequencing using the Illumina HiSeq platform, GBS was successfully employed to produce a high density, saturated linkage map for a red raspberry (*R. ideaus*) mapping population. Problems of missing data and false negative genotyping calls were overcome by relying on data from SNP genotyping bins to perform imputation of missing and erroneous data points within the segregation data matrix using Maskov. The ‘Heritage’ and ‘Tulameen’ linkage maps produced were of a comparable length to previously-published linkage maps of the species [4,47] and to the linkage maps of closely-related genera such as diploid *Fragaria*[3,11] and diploid *Rosa*[48], but shorter than the L×GM *Rubus* linkage maps published by Graham et al. (2006, 2011) [42,45]. A comparison of common SSR markers revealed almost complete colinearity between the ‘Heritage’ and L×GM maps, but a reduction in genetic distance on the ‘Heritage’ map. Since the process of imputation employed tended towards conservatively placing markers into genotypic bins and thus eliminating the occurrence of spurious double recombination events within the data, the process would also tend to reduce the overall length of the linkage maps produced. However, positioning of common markers has demonstrated that the imputation process employed results in accurate marker placement, albeit at the expense of precise marker ordering within genotyping bins.

Despite the calculation of relatively low inbreeding coefficients for both ‘Heritage’ and ‘Tulameen’ (0.094 and 0.069 respectively) by Dale et al. (1993) [49], in this investigation we observed almost twice the level of heterozygous SNPs in the genome of ’Heritage’ than in the genome of ‘Tulameen’. Relatively high levels of genome differentiation and heterozygosity is a feature of red raspberry germplasm, despite the majority of modern varieties being derived from a narrow genetic base [49]. The genome of ‘Heritage’, the more heterozygous of the two parental genotypes, is currently being sequenced by an international consortium [50], and thus data from the relative positions of SNPs mapped in this investigation within sequence scaffolds of the ‘Heritage’ genome sequence will help to validate the SNP positioning on the H×T linkage maps and will increase the precision of SNP positions within genotype blocks. Additionally, the genetic positions of the SNPs on the H×T linkage maps will permit anchoring of sequencing scaffolds and the development of pseudochromosomes for the *Rubus* genome sequence, as had been performed for other highly heterozygous genome sequences [6,15].

The GBS approach used in this investigation enabled the identification and mapping of an unprecedented number of sequence characterized markers in *Rubus* and to produce the most saturated linkage map for a species within the Rosaceae family to date, at a fraction of the time and cost of developing maps for *Rubus* using traditional marker technologies such as SSRs [4] and gene specific and EST-based markers [45]. Indeed, the methods employed here are more cost-effective than the array-based methods of SNP detection and scoring, such as the IRSC Infinium whole genome genotyping array recently developed and used for linkage map construction in *Malus*[22,26]. However, GBS as used in this investigation yielded data containing large amounts of missing values. Splitting the library preparations between two lanes of sequencing allowed examination of the effect that varied quality in sequencing has on the outcome. One sequencing center provided data with nearly twice as many uncalled bases and in the current implementation of Stacks reads containing uncalled bases are discarded. Increasing depth of coverage by sequencing each individual in multiple lanes would likely resolve the issue of missing values, but it is also expected that starting with DNA of increased quality and purity would result in a more uniform restriction digestion and adapter ligation. Therefore performing manual DNA extraction or preparing multiple libraries with independent automated DNA extractions may result in more uniform sequencing and fewer missing values when the GBS method of Elshire et al. (2011) [37] is applied to linkage map construction. The most robust method is likely to be one in which two independent library preparations are conducted and sequenced for each progeny individual in separate lanes. Choosing an enzyme that cuts less frequently could also reduce the number of missing values by increasing coverage per restriction fragment. Using a more rare cutting enzyme could also potentially reduce the amount of sequenced chloroplast DNA. However, the use of rare cutting enzymes in pseudo-testcross progenies that are less heterozygous would also dramatically decrease the number of markers detected in the AA × AB and AB × AA configuration. As sequencing yield and quality continues to increase and costs continue to decrease, the desire to conduct larger and more highly multiplexed experiments may propagate the problem of missing data further. The Maskov imputation program that we present here can be used to overcome the challenges of missing data through map-based imputation.

On previously reported *Rubus* linkage maps, regions of significant segregation distortion have been observed [4]. Similar regions of segregation distortion were observed in this investigation, however, the depth of marker saturation of the linkage maps presented here allowed us to plot the occurrence of segregation distortion along each linkage group with a high degree of precision. A number of well-defined regions of the ‘Heritage’ and ‘Tulameen’ linkage maps exhibited significant segregation distortion and in many cases these regions were conserved between the two parental linkage maps, indicating the presence of lethal or sub-lethal genes that are conserved in heterozygous form in both parental genotypes. Jennings (1967) [51] reported on the genetics of two loci, *H* conferring the presence of cane pubescence, and *T* conferring the presence of red pigmentation, and observed that they are rarely present in the homozygous forms *HH* and *TT* which was postulated to be due to lethal or sub-lethal genes linked in coupling to the dominant allele of each gene. Later, a gene affecting the viability of seeds in raspberry progenies and determining the presence or absence of cotyledonary glands was also described by Jennings (1972) [52]. Graham et al. (2006) [42] reported a genetic map position for gene *H* on LG2 of the ‘Latham’ × ‘Glen Moy’ genetic linkage map, which is within the region of one of the defined areas of segregation distortion on the ‘Heritage’ linkage map, as well as on the linkage map of Sargent et al. (2007) [4], but not on the ‘Tulameen’ map. Both ‘Heritage’ and ‘Tulameen’ present the glabrous recessive phenotypes for gene *H* (i.e. *hh*) however, the maps presented here suggest that there are a number of genes distributed throughout the seven *R. idaeus* chromosomes that exhibit a lethal or highly detrimental sub-lethal effects which are conserved in heterozygous form in the ‘Heritage’ and ‘Tulameen’ genotypes, presumably due to advantages associated with pleiotrpic effects of the recessive lethal or sub-lethal alleles. These genes are most likely a factor in the high degree of heterozygosity that is maintained in *Rubus* despite the breakdown of the self-incompatibility system in the species [53]. Whilst segregation distortion has previously been observed on genetic linkage maps of *Rubus*[4], in this investigation, we have mapped markers in sufficient numbers to permit the identification of a number of conserved genetic regions between linkage maps putatively responsible for biased transmission of alleles. The availability of a genome sequence for *Rubus* would potentially allow the identification of candidate genes creating the segregation distortion apparent on the H×T linkage maps.

## Conclusions

Using GBS followed by imputation of missing data guided by marker membership to genotyping bins using Maskov, we have identified and mapped a total of 6912 SNPs in *Rubus* and developed a comprehensive SNP reference map for red raspberry. As the flanking sequences of each of the SNPs presented here have been defined and are available in Table S2, marker positions from this investigation can be used to inform studies in other *Rubus* populations. Fine mapping of regions of interest could be performed either through development of CAPs markers [25], or HRM assays [24] from SNPs within regions of interest to saturate existing *Rubus* linkage maps, or by first identifying heterozygous SNPs from GBS of parental lines of genetically undefined mapping populations. This could be followed by design of assays for selected heterozygous SNPs distributed throughout the seven *Rubus* linkage groups. The approach described here is suitable for the rapid and reliable development of saturated linkage mapping resources for any organism, whether or not it has been previously genetically characterized, or has an available genome sequence, and provides a wealth of genetic information that can serve as the starting point for downstream genetic investigations such as QTL analyses, positional cloning of genes controlling traits of interest, the anchoring of genome sequence contigs and the development of genomic selection strategies.

## Methods

### Plant material and DNA extraction

To generate a segregating population, a cross was made between the *R. idaeus* cultivars ‘Heritage’ (National Clonal Germplasm Repository accession # PI 553382) and ‘Tulameen’ (National Clonal Germplasm Repository accession # PI 618441). The resulting seeds were germinated and grown under glasshouse (double walled polycarbonate) conditions and the population denoted H×T for ease of reference. Young fresh leaf material was collected from the progeny, snap frozen and ground to a fine powder under liquid nitrogen. DNA was extracted in 96-well plate format using the Omega-E-Z extraction kit according to the manufacturer’s recommendations. DNA was quantified using PicoGreen (Invitrogen) against a λ standard DNA dilution series with a Synergy 2 fluorimeter (BioTek) then stored at −20°C prior to sequencing.

### Genotyping by sequencing

To determine the optimal concentration of sequencing adapter to use per unit of DNA, a titration was performed using the methods, barcodes, adapters, and primers of Elshire et al. (2011) [37]. Briefly, eight titrations were performed with 200 ng of DNA from ‘Heritage.’ DNA was digested with *Ape*KI (New England Biolabs, Ipswitch MA) for 2 hours at 75°C. Following digestion, various quantities of *Ape*KI adapter (1.8 ng, 2.4 ng, 3.6 ng, 4.2 ng, 4.8 ng, 5.4 ng, 6.0 ng, and 7.2 ng) were ligated to the resulting restriction fragments using T4 ligase (New England Biolabs, Ipswitch, Massachusetts, USA) with 60 minute incubation at 22°C followed by a 30 minute ligase denaturation step at 65°C. The ligation reaction was purified with a Qiagen PCR cleanup kit (Qiagen, Valencia, California, USA) as per the manufacturer’s instructions.

Next, 10 μl of the purified reaction was used in a 50 μl PCR reaction with 25 μl PCR 2x Taq Master Mix (New England Biolabs, Ipswitch, Massachusetts, USA), and 25 pmol of each primer. Thermal cycling was initiated with 5 minutes at 72°C and 30 seconds at 98°C followed by 18 cycles of 10 seconds at 98°C, 30 seconds at 65°C and 30 seconds at 72°C. A final extension was performed at 72°C for 5 minutes. An additional Qiagen PCR cleanup was performed and the subsequent libraries were analyzed on an Agilent Bioanalyzer (Agilent Technologies, Santa Clara, California, USA) and the electropherograms examined for library and dimer peaks. The adapter concentration of 3.6 ng yielded a satisfactory library without adapter dimer or other highly aberrant peaks. Thus genotyping by sequencing was performed as in the titration with the exception that 100 ng of DNA from progeny and correspondingly 1.8 ng of uniquely bar-coded adapter was used for each sample. All reactions were performed in separate wells for each genotype from the population and were pooled after ligation and before a 25 μl PCR. Digestion, ligation PCR conditions, and thermal cycling were the same as in the titration.

The 71 progeny from the H×T population were split between two library preparations (42 genotypes in the first and 29 genotypes in the second) and sequenced independently at two different sequencing centers, both as part of 96-plex reactions. Libraries were sequenced on the Illumina HiSeq 2000 sequencing platform (Illumina, San Diego, California). Sequencing reads were subsequently processed with custom perl scripts [54]. Furthermore the script trimmed reads to 64 nt and only reads with *Ape*KI restriction sites were retained. Data was further processed in Stacks [46] with Stacks de novo, default settings and automated genotype corrections were allowed.

Quantification of over-represented reads and unique reads was determined by counting read frequency with a custom UNIX shell script. Reads with frequency greater than 1000 were initially screened against the NCBI nt database. After the initial determination that many over represented reads were highly similar to other chloroplast sequences, all overrepresented reads were aligned to the *Fragaria vesca* chloroplast genome (GenBank: JF345175.1) using Bowtie [55] with default settings for reads in FASTA format.

### Microsatellite amplification and scoring of heterozygous markers

The fingerprinting set proposed by Fernández-Fernández et al. 2011 [8] was used to confirm the parentage of the seedlings and to identify those resulting from uncontrolled outcrossing or selfing. Seedlings resulting from outcrossing were removed from further analysis. Additionally, selected primer pairs from published primer sets [39-43] were labelled on the forward primer with either 6-FAM or HEX fluorescent dyes (IDT, Belgium) or NED and PET (Life Technologies Corporation, Carlsbad, California, USA) and tested for heterozygosity in the parental genotypes ‘Heritage’ and ‘Tulameen’ in single PCR reactions. From these, heterozygous markers from each of the seven previously reported *Rubus* linkage groups were identified for scoring in the full H×T progeny. Primer pairs generating heterozygous amplicons in the parental genotypes were combined by product size and fluorescent dye colour into multiplexes of up to eight primer pairs and PCR was performed using the ‘Type-it’ PCR mastermix (Qiagen, Valencia, California, USA) following the manufacturer’s recommendations, in a final volume of 12.5 μl. Reactions were performed using the following PCR cycles: an initial denaturation step of 95°C for 5 min was followed by 28 cycles of 95°C for 30 s, an annealing temperature of 55°C decreasing by 0.5°C per cycle until 50°C for 90 s and 72°C for 30 s, followed by a 30 min final extension step at 60°C. PCR products were fractionated by capillary electrophoresis through a 3100 genetic analyser (Life Technologies Corporation, Carlsbad, California, USA). Data generated were collected and analysed using the GENESCAN and GENOTYPER (Life Technologies Corporation, Carlsbad, California, USA) software.

### Manual imputation of genotyping by sequencing data for linkage map construction

Sequence data obtained for any one individual contained both missing data and a degree of false-negative SNP calls. These missing data and false negatives resulted from library construction, the relatively low depth of sequencing coverage per progeny individual due to multiplexing, and sequencing biases created by the sequencing platform employed in this investigation, following initial marker ordering. Missing data complicated the computation of reliable marker ordering and sequencing artifacts led to a high number of perceived double recombination events per individual following initial map construction. Thus for accurate and reliable linkage map construction using GBS data, we implemented a system of data imputation that increased the accuracy of marker placement, at the expense of precision in localised marker order. Data output from Stacks [46] was combined with SSR data and formatted for linkage mapping using the standard codes for a ‘cross pollination’ (CP) type progeny of JOINMAP 4.0 (Kyasma, NL).

### Imputation Rationale

**Figure 5:**
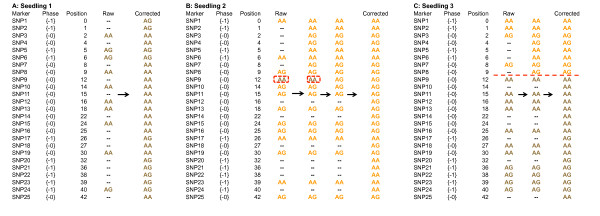
Rationale for the imputation of missing values from genotyping by sequencing (A) imputation of missing values in the same genotype/phase configuration where no recombination is observed; (B) imputation where a false negative genotype is observed; (C) imputation where a change in phase/genotype configuration is observed following an observed recombination event.

Markers were initially ordered along the seven linkage groups from each parent of the H×T mapping progeny using the maximum likelihood algorithm of JOINMAP 4.0 (Kyasma, NL) to provide a map position based on the raw segregation data obtained from Stacks. Following initial ordering, marker positions were individually scrutinized by eye to ensure a broadly correct map position and the determination of marker genotype blocks. Imputation of missing data and genotyping errors was then performed using the following rationale: Markers were colour-coded according to genotype and phase and genotypes of each individual in the progeny were scrutinised one-by-one beginning at the ‘proximal’ end of each linkage group. Recombination events were taken to be rare events in any given chromosome of any given individual, so when two or more genotypes coded in the same phase and genotype configuration (i.e. in the same colour) were interspersed by one or more missing values, those values were taken to have the same phase and genotype configuration as the flanking genotypes, i.e. the assumption was that no recombination had occurred between the markers for which data were missing, and their values were imputed (interpolated) according to the calculated phase of the marker (Figure 5A). When a marker genotype in the opposite phase\genotype configuration was encountered, the genotypes following the marker were scrutinised. If the following marker displayed the opposite phase\genotype configuration, the previous marker genotype was assumed to be erroneous and its genotype was amended to fit with flanking markers in the genotype block (Figure 5B). If however the following genotypes were in the same phase and genotype configuration, a recombination was assumed to have occurred and missing values between subsequent markers were imputed with the corresponding phase and genotype configuration as appropriate (Figure 5C) to create a new marker genotype block.

### Automated imputation of genotyping by sequencing data for linkage map construction

#### The maskov algorithm

Initial output from marker ordering with the maximum likelihood algorithm of JOINMAP 4.0 (Kyasma, NL) is encoded according to genotype phase and configuration as 1 (AB phase {1-} and AA phase {0-}), 0 (missing values), and −1(AA phase {1-} and AB phase{0-}). The imputation algorithm corrects errors and missing values using a three pass process. The first pass temporarily removes all missing values because they do not contain information and reduce accuracy of recombination edge detection. Pass two identifies recombination edges by convolving each genotype with a mask vector according to the following formula:

(1) $$\mathrm{convolved}\left(x_{i}\right)=\sum_{k=-E}^{E}\mathrm{genotype}\left(x_{i+k}\right)\times \;M_{E+k}$$

where M, the convolution mask, is a vector of size 2*E*+1, genotype (*x*) is the original data set with errors (but without missing values), and *convolved(x)* is a function that describes the location of recombination events.

The coefficients of the mask vector M are weighted to compute the effective first derivative at each point. The location of an edge is defined as any position *x*_*i*_ where |*convolved*(*x*_*i*_)| > *E* and where *E* is a user defined parameter that represents the maximum number of expected consecutive errors. The sign of *convolved(x*_*i*_*)* decides which phase transition is detected. The third and final pass fills in the “blocks” between the recombination edges using “winner take all” criteria to correct errors. As the length of the mask vector *M* increases, recombination edges are detected more reliably but the exact location of an edge will become less accurate in the presence of errors. Maskov also has a user-defined parameter that controls the amount of tolerated missing data for a given genotype and a threshold value *T* (by default *T*= *E*) that controls the detection of recombination edges. Additional description of the algorithm, including usage information and screenshots are provided (Additional file 7: Text S1**)**. The first release of the program called Maskov (Version 1.01) is freely available at the Maskov google group.

#### Automated imputation with Maskov in *Rubus idaeus*

Maskov 1.01 was used to visualize recombination events and to perform imputation with the initial output from marker ordering with the maximum likelihood algorithm of JOINMAP 4.0 (Kyasma, NL). Imputation parameters in Maskov were set to *E* = 5 with the default threshold of *E*, and the maximum amount of missing data set at seventy percent.

### Linkage map construction

Imputed GBS data along with data generated for SSR loci were analysed using the maximum likelihood function of JOINMAP 4.0 (Kyasma, NL) to enable linkage map construction. Data were grouped using a minimum LOD score of 7.0 and maps were constructed using the default maximum likelihood parameters. Following initial linkage map construction, the markers were colour-coded according to phase and genotype as previously described, and marker positions were visually inspected and resolved where necessary. Markers with identical genotypes in the H×T progeny were grouped into mapping bins of identical genotypes and a single genotype for each bin was then used for final map construction using regression mapping in JOINMAP 4.0 (Kyasma, NL) applying the Kosambi mapping function. Marker placement was determined for each linkage group of the two parental maps using a minimum LOD score threshold of 7.0, a recombination fraction threshold of 0.35, ripple value of 1.0, jump threshold of 3.0 and a triplet threshold of 5.0. Linkage groups were compared to previously published maps using the positions of common SSRs markers and nomenclature follows the recently revised numbering system of Bushakra et al. (2012)[44]. Maps presented were plotted using MapChart 2.0 [56].

### Distribution of segregation distortion across the H × T linkage maps

Segregation distortion was determined by calculating *x*^*2*^ values for all mapped markers using JOINMAP 4.0 (Kyasma, NL). Relative distortion along each linkage group of H×T maps was determined by plotting *x*^*2*^ values against marker positions along each linkage group. Significance thresholds for P=0.05, P=0.01 and P=0.001 were plotted as dashed lines on the graphs.

## Abbreviations

GBS: Genotyping by Sequencing; SSRs: Simple Sequence Repeats; RFLPs: Restriction fragment length polymorphisms; CAPs: Cleaved amplified polymorphic DNA; SNPs: Single nucleotide polymorphisms; HRM: High resolution melting; RAD: Restriction-site associated DNA; QTL: Quantitative trait loci; PCR: Polymerase chain reaction; LG: Linkage group; CP: Cross pollination; LOD: Logarithm (base 10) of odds.

## Competing interests

The authors declare that they have no conflicts of interest.

## Authors’ contributions

JAW conceived of the study. JAW performed the GBS library preps and associated bioinformatics. DJS generated linkage maps. JB programmed Maskov and conceived of the convolution solution. JB, JAW, and DJS contributed to the Maskov algorithm development. JAW and DJS performed data analysis. FFF ran SSRs. PM made the crosses and cared for plant material. CAW, NB, FFF, PM, JDS, RV, and RV contributed resources. JAW, JB and DJS wrote the paper. The manuscript was read, edited, and approved by all authors.

## Supplementary Material

Additional file 1: Figure S1 The per base phred scores for Library One and Library Two showing the mean phred score (the point) and the upper and lower quartiles (the ends of lines). Click here for file

Additional file 2: Figure S2 The per read phred scores and their frequencies for Library One and Library Two. Click here for file

Additional file 3: Figure S3 The number of sequencing reads versus the number of unique sequencing reads for Library One and Library Two. Click here for file

Additional file 4: Table S1 The count of over-represented sequencing reads, their position of alignment against the *Fragaria vesca* chloroplast genome (GenBank: JF345175.1), and the position of SNPs between the chloroplast sequences. (XLSX 53 kb) Click here for file

Additional file 5: Figure S4 Percent missing values as a function of the number of sequencing reads per genotype. Click here for file

Additional file 6: Table S2 The markers mapped to the two parental linkage maps of the H×T mapping progeny, including the linkage group and map position to which they were located, the SNP type and the nucleotide sequence flanking each side of each SNP. Click here for file

Additional file 7: Text S1 The manual and additional descriptions of the imputation algorithm (Maskov). Click here for file
