# Supplementary figures and images for: Saturated linkage map construction in Rubus idaeus using genotyping by sequencing and genome-independent imputation
Source: BMC Genomics. 2013 Jan 16;14:2. doi: 10.1186/1471-2164-14-2 (PMC3575332; doi:10.1186/1471-2164-14-2)

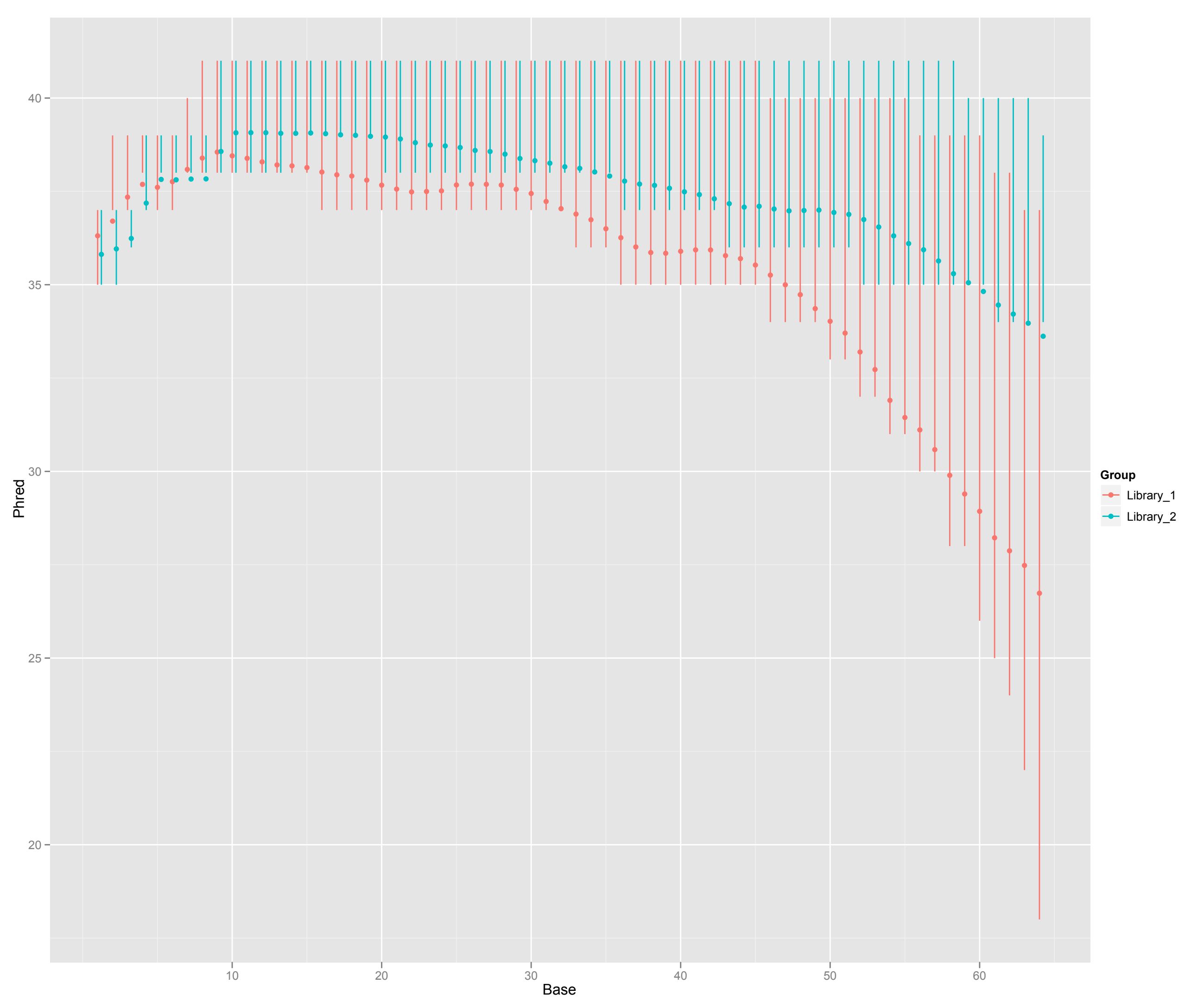

Supplement: Additional file 1: Figure S1 — The per base phred scores for Library One and Library Two showing the mean phred score (the point) and the upper and lower quartiles (the ends of lines). [file 1471-2164-14-2-S1.pdf]

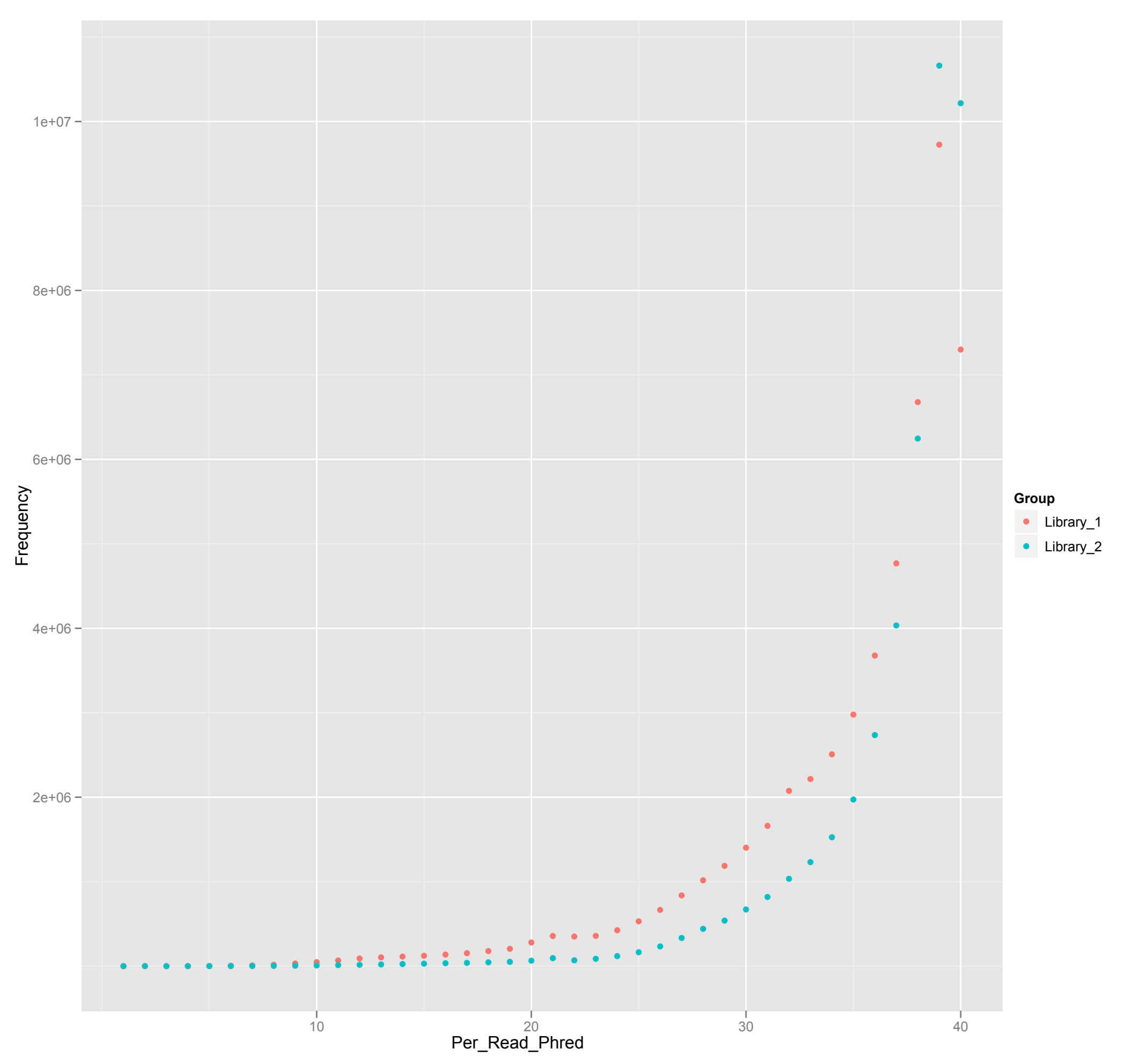

Supplement: Additional file 2: Figure S2 — The per read phred scores and their frequencies for Library One and Library Two. [file 1471-2164-14-2-S2.pdf]

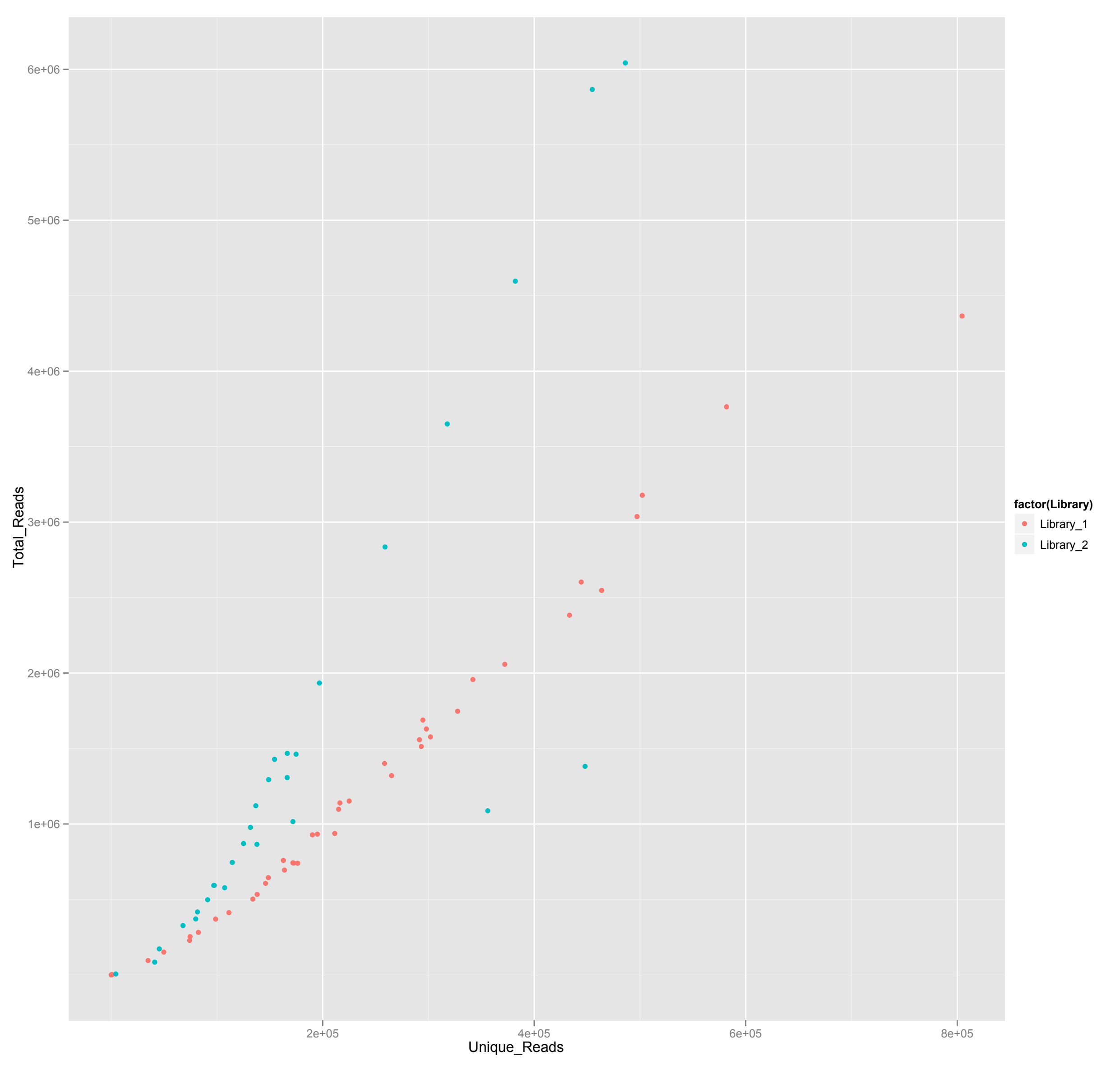

Supplement: Additional file 3: Figure S3 — The number of sequencing reads versus the number of unique sequencing reads for Library One and Library Two. [file 1471-2164-14-2-S3.pdf]

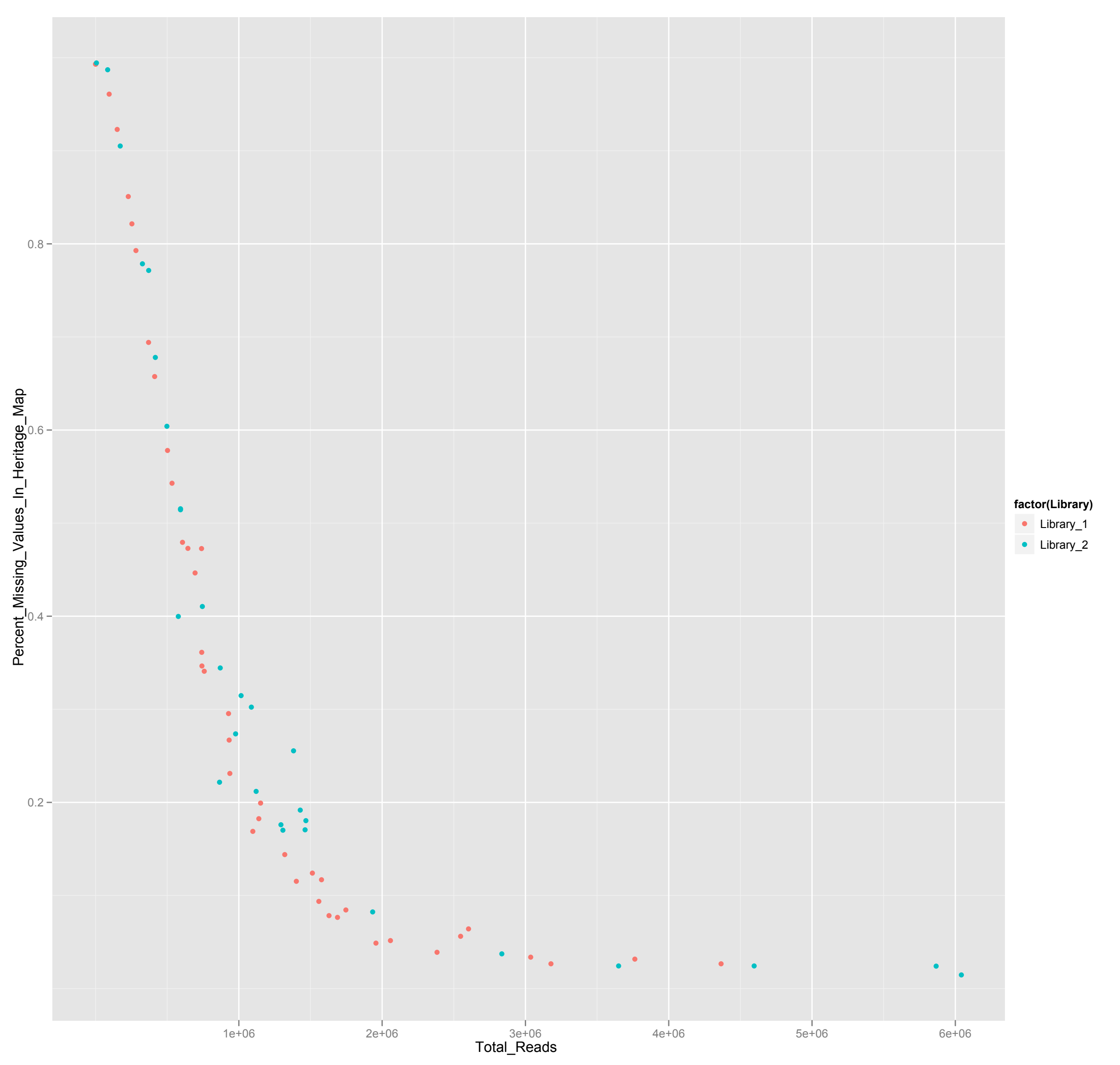

Supplement: Additional file 5: Figure S4 — Percent missing values as a function of the number of sequencing reads per genotype. [file 1471-2164-14-2-S5.pdf]
