# Supplementary material for: Saturated linkage map construction in Rubus idaeus using genotyping by sequencing and genome-independent imputation
Source: BMC Genomics. 2013 Jan 16;14:2. doi: 10.1186/1471-2164-14-2 (PMC3575332; doi:10.1186/1471-2164-14-2)
Supplement: Additional file 7: Text S1 — The manual and additional descriptions of the imputation algorithm (Maskov). [file 1471-2164-14-2-S7.pdf]

Usage

Maskov is written in Java and can run on Mac OS X, Linux, and Windows operating systems as long as Java 1.6 or later is installed and operational. The program is launched either by clicking on the supplied jar file or by executing the command `java -jar Maskov.jar` from the terminal. If launching the program from the command line the user may need to supply the full path to both Java 1.6 (or later) and to the `Maskov.jar` file depending on user specific system defaults.

Once Maskov is launched the user simply opens a single recombination group file in Joinmap format after maximum likelihood ordering. Maskov performs imputation rapidly after opening the file and results are visualized by clicking on a column value (Note that the derivative function is limited in the visualizations to the threshold value for ease of interpretation and plotting). When imputation parameters controlling the mask size, derivative thresholds, and percent missing data allowable are altered the data matrix is automatically recomputed. Columns with more missing data than the missing data threshold are corrected to all missing and are highlighted in red. The resulting imputed data set can be exported in Joinmap format or in a compressed numerical representation by clicking the toggle switch to the far right of the control panel. The user can also choose to export a single marker per bin or the complete set by selecting the appropriate save button. If desired, a configuration file is placed in the directory where the program was launched and will set default parameters for imputation as well as the path to the directory containing the files for imputation.

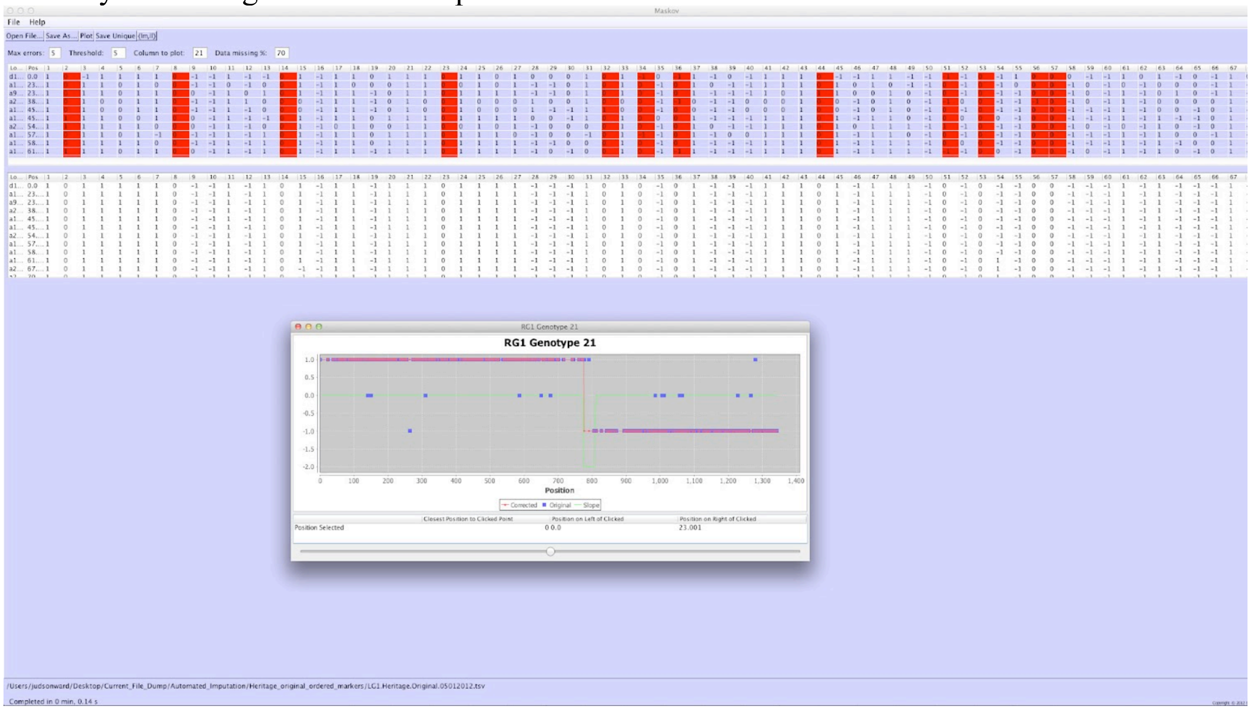

Algorithm Outline

Given a series of column data $PM_{\text{column}} = \{(x, f(x)) \mid x \in \Re^+, f(x) \in \{-1,\ 0,\ 1\}\}$ that includes positions with error values and missing values (missing values are those where $\mathrm{f}(\mathrm{x}) = 0$), produce a corrected series of data $PM'_{column} = \{(x, g(x)) \mid x \in \Re^+, g(x) \in \{-1,\ 1\}\}$ where g is a transformation of f that corrects error and missing data with the goal of minimizing the number of transitions (a transition occurs when $g(x_i) \neq g(x_{i+1})$). $\Re^+$ represents the set of positive real numbers.

1. Data Conditioning step

Missing data degrades the performance of the convolution process. Before trying to resolve errors, temporarily remove all instances of missing data. The convolution will have fewer errors to resolve and therefore perform better.

Assumption: $\text{max block length} \gg \text{max error sequence length}$

Note that any edge detection technique will give poor results if there are a large number of errors or missing data near the edge of a block.

2. First Derivative step

Given a mask vector M with elements M_a

Approximate the first derivative of f by $f'(x_i) = \sum_{k=-E}^{E} f(x_{i+k}) \cdot M_{E+k}$

Note that $f'(x) \in \mathbb{Z}$ and block transitions will appear in $f'(x)$ as non-zero values. $\mathbb{Z}$ represents the set of Integers.

E is a configurable parameter representing the maximum number of sequential errors to resolve (ie. the maximum number of sequential errors expected)
M is a convolution mask of $2E+1$ elements assigned in the following pattern to react to transitions between blocks in the presence of errors:

$M = [M_{-E}, M_{-E+1}, ..., M_0, ..., M_{E-1}, M_E]$ where $M_{-i} = -1$, $M_0 = 0$, $M_i = 1$

For example, if $E = 3$ then $M = [-1, -1, -1, 0, 1, 1, 1]$

As E increases, M will detect edges through longer sequences of missing data and errors at the expense of accuracy of the exact transition location.

A note about the linear step model for M:

> To correct errors in continuously valued functions, the elements of M are usually chosen from a ***normal*** distribution. A normal mask computes a weighted average of neighboring values and works well for continuous data because it favors closest neighbors that are most likely closest to the correct value.
>
> But f is a discontinuous step function over a range of only two values. Our ***step*** distribution of M essentially computes the modal average of all nearby values, which is more appropriate for a discrete valued function over a small range.

3. Error Correction step

Now, define a filter $t(x)$ to remove false transitions based on $f'(x)$ and a configurable threshold T

$$t(x) = \begin{cases} 1 : f'(x) > T \\ -1 : f'(x) < T \\ 0 : otherwise \end{cases}$$

False transitions are triggered by data errors and therefore will be weakly indicated by a small first derivative. The threshold T will remove them.

Generate artificial endpoint values to handle the first and last blocks correctly:

$$t(0) = -t(x_{first}), t(x_{\max}) = -t(x_{last})$$

where

x_{first} and x_{last} are the smallest and largest values where $t(x) = 0$, respectively.

Now define $g(x)$ based on t:

$$g(x) = \begin{cases} 1 : a < x \le b, t(a) = 1, t(b) = -1 \\ -1 : a < x \le b, t(a) = -1, t(b) = 1 \end{cases}$$

Programming Details and Data Structures

Given M, a list of markers with each marker (“row”), Mi, consisting of a position, phase and a set of configuration values, one for each individual. The configuration values can be viewed as “columns” in the file to examine a single individual. Using JoinMap file terminology, M has `NLOC` markers (rows) and defines `NIND` configuration values (columns) even though the exact value of `NLOC` is not given in advance.

```
Marker {
     double          position
     integer         phase
     string[NIND]    config     // Array: number of columns is known
}

List<Marker>         M    // List: number of rows is unknown
```

Valid values for the `config` string are “xy”,”wz”, or ”—“ for missing data. Valid values for `phase` are 0 and 1.

Our analysis depends on the order of rows but not the columns. But now we know the number of rows so we can transform M to CM (a set of columns). This lets us process each individual independently, using the relationship between rows to correct errors and missing data. CM is fixed size because the number of rows is known after reading the entire input.

First, convert the list to a new data structure, `Config`, that supports column processing.

```
Config {
     double          position
     integer         phase
     string          config     // No longer an array at this level
}

Config[NLOC]         MarkerConfigs   // The array is now here
MarkerConfigs[NIND]  CM
```

Combine the phase and configuration into one ternary valued result, ***Ind***, according to the following table. Then simplify `Config` to a new data structure, `PhasedGenotype`, for the new result.

IND		**phase**	
		**1**	**0**
**config**	**xy**	-1	1
	**wz**	1	-1
	**--**	0	0

```
PhasedGenotype {
     double          position
     integer         ind        // -1, 0, or 1
}

PhasedGenotype[NLOC] PhasedMarkers
PhasedMarkers[NIND]  PM
```

Before computing first derivatives, remove the missing data (i.e. ind=0) because they may unreliably appear to be transitions and store the result into a new `ConditionedGenotype` data structure. Each column can no longer be defined as an array because we can't predict how many entries will remain after the zeroes are removed.

```
ConditionedGenotype {
     double          position
     integer         coniditionedInd      // Only -1 or 1
}

List<ConditionedGenotype> ConditionedMarkers
ConditionedMarkers[NIND]       RM
```
